# Degos Disease (Malignant Atrophic Papulosis) With Granular IgM on Direct Immunofluorescence

**DOI:** 10.7759/cureus.12677

**Published:** 2021-01-13

**Authors:** Tatsiana Pukhalskaya, Julia Stiegler, Glynis Scott, Christopher T Richardson, Bruce Smoller

**Affiliations:** 1 Pathology, University of Rochester School of Medicine and Dentistry, Rochester, USA; 2 Dermatology, University of Rochester School of Medicine and Dentistry, Rochester, USA; 3 Dermatopathology, University of Rochester School of Medicine and Dentistry, Rochester, USA; 4 Pathology and Dermatology, University of Rochester School of Medicine and Dentistry, Rochester, USA

**Keywords:** degos disease, malignant atrophic papulosis, cutaneous lupus erythematosus

## Abstract

Degos disease is a rare vasculopathy characterized by skin papules with central porcelain white atrophy and a surrounding telangiectatic rim. Etiology of this condition is unknown. There are benign and systemic forms of the disease, and the latter may lead to fatality. Connective tissue diseases with Degos-like features have been described, and many authors speculate that Degos is not a specific entity but, rather, a distinctive pattern of disease that is the common endpoint of a variety of vascular insults.

We describe the case of a 45-year-old female who presented with numerous red papules with sclerotic white centers and minimal systemic symptoms. Laboratory workup was notable for a negative autoimmune panel and hypercoagulation panel. Histopathology revealed epidermal atrophy, abundant dermal mucin, a perivascular lymphocytic infiltrate, interface inflammation, papillary dermal hemorrhage, and several small thrombi in the mid-to-superficial vessels. Direct immunofluorescence (DIF) showed strong granular immunoglobulin M (IgM) deposition at the dermal-epidermal junction. Based on the pathognomonic skin findings, persistently negative antinuclear antibody, lack of systemic signs of systemic lupus erythematosus, and characteristic hematoxylin and eosin findings, a diagnosis of Degos disease was rendered.

In the fewer than 200 published cases of Degos disease, DIF findings have been conflicting and often negative. The DIF pattern of granular IgM is classically found in lupus erythematosus. To our knowledge, this is the first case of Degos disease reporting deposition of strong granular IgM on DIF. This case serves as additional evidence of the considerable clinical and histologic overlap between Degos disease and lupus erythematosus.

## Introduction

Malignant atrophic papulosis (MAP), also known as Degos disease, is a rare and potentially fatal vasculopathy of unknown pathophysiology. It was first described by Kohlmeier in 1941 and documented as a distinct entity by Degos et al. in 1942. Fewer than 200 cases have been reported since [1]. The first manifestation of the disease typically occurs between the second and fifth decades of life, but can occur at any time. Cases of MAP have been reported in various clinical scenarios including following streptococcal infection, during pregnancy, and in a patient with acquired immunodeficiency syndrome [2-5]. There are also several reports of familial involvement [6,7].

MAP has benign (skin-limited) and malignant (systemic) forms. Skin lesions are present in the different stages of evolution. Initially, crops of small 0.5-1.0 cm erythematous papules appear, most commonly on the trunk and upper extremities. The papules slowly evolve to become umbilicated with porcelain white central atrophy and a telangiectatic rim. The malignant form is associated with infarcts in the gastrointestinal tract and central nervous system and may result in fatality. Involvement of other organs has been reported [8-11]. The skin findings may precede the development of systemic ischemic events by years.

There is a wide spectrum of histologic findings in cutaneous lesions of Degos disease. The most prominent features are epidermal atrophy, hyperkeratosis, and a wedge-shaped area of dermal necrosis. A perivascular lymphocytic infiltrate may be present at the edge of the necrotic area. There may also be thrombi in small vessels, dermal mucin, and changes that resemble lupus-like panniculitis. Basal vacuolar change is also seen in some cases. This histological picture creates speculation that Degos disease is not a specific entity but, rather, a distinctive pattern of disease that is the common endpoint of a variety of vascular insults [12].

## Case presentation

**Figure 1:**
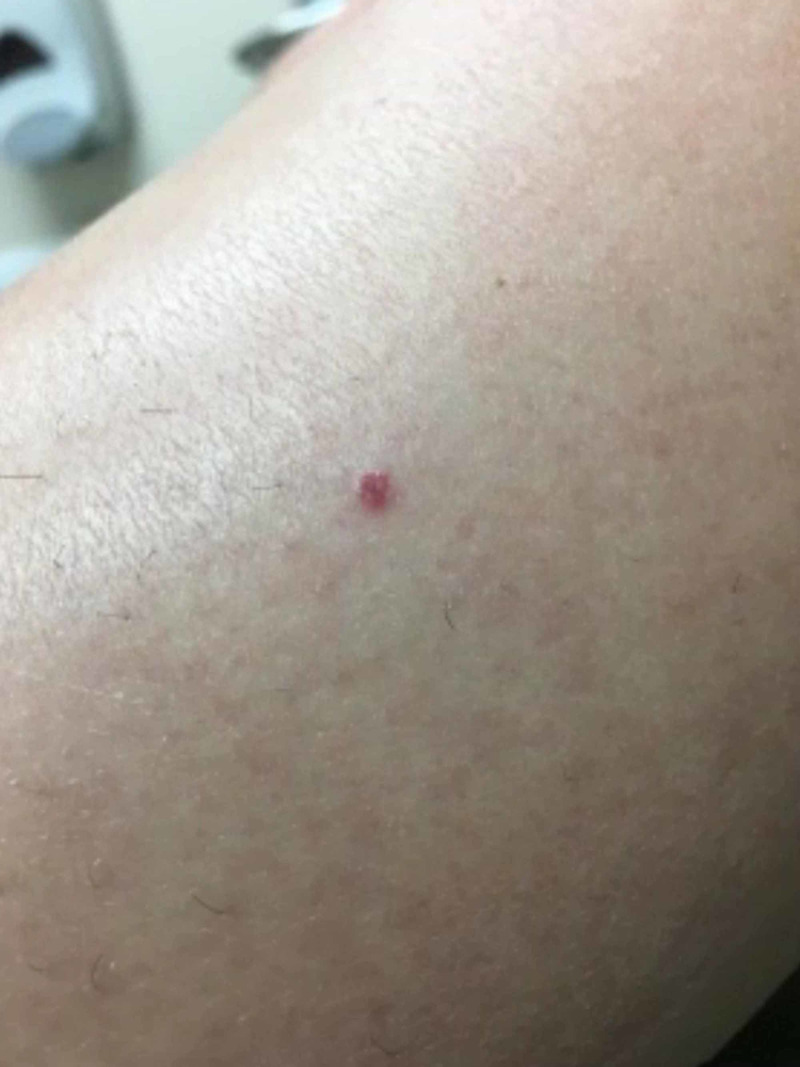
Clinical illustration of the lesion that presented as a small red papule.

A 46-year-old female presented to the dermatology clinic with a seven-month history of asymptomatic pink-to-violaceous macules and papules predominantly on the legs, with a few lesions on the abdomen and left ventral forearm. The lesions were 1-2 mm in diameter and showed a variety of different morphologies (Figure 1).

**Figure 2:**
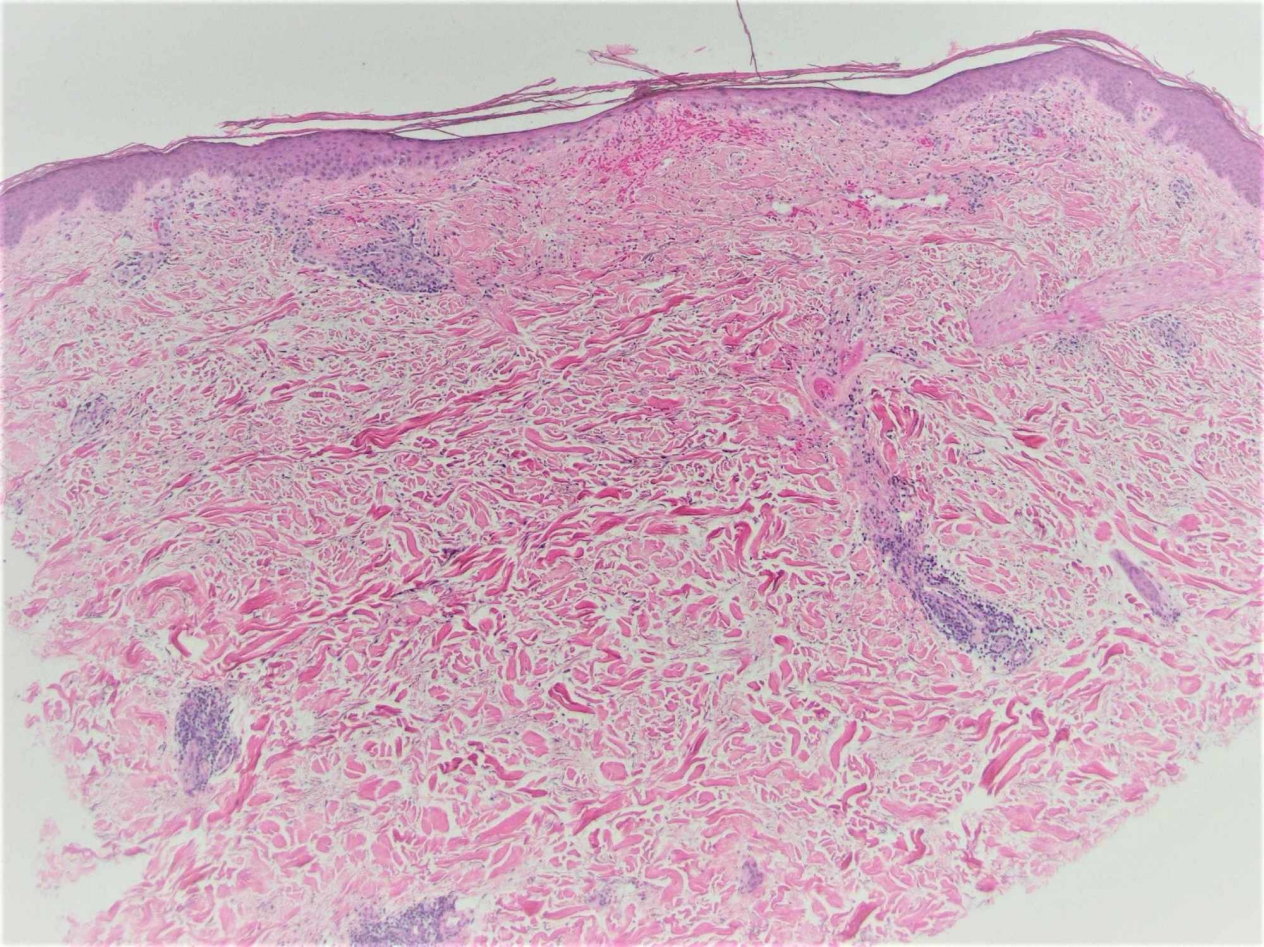
Punch biopsy of the resolving lesion (H&E ×40).

**Figure 3:**
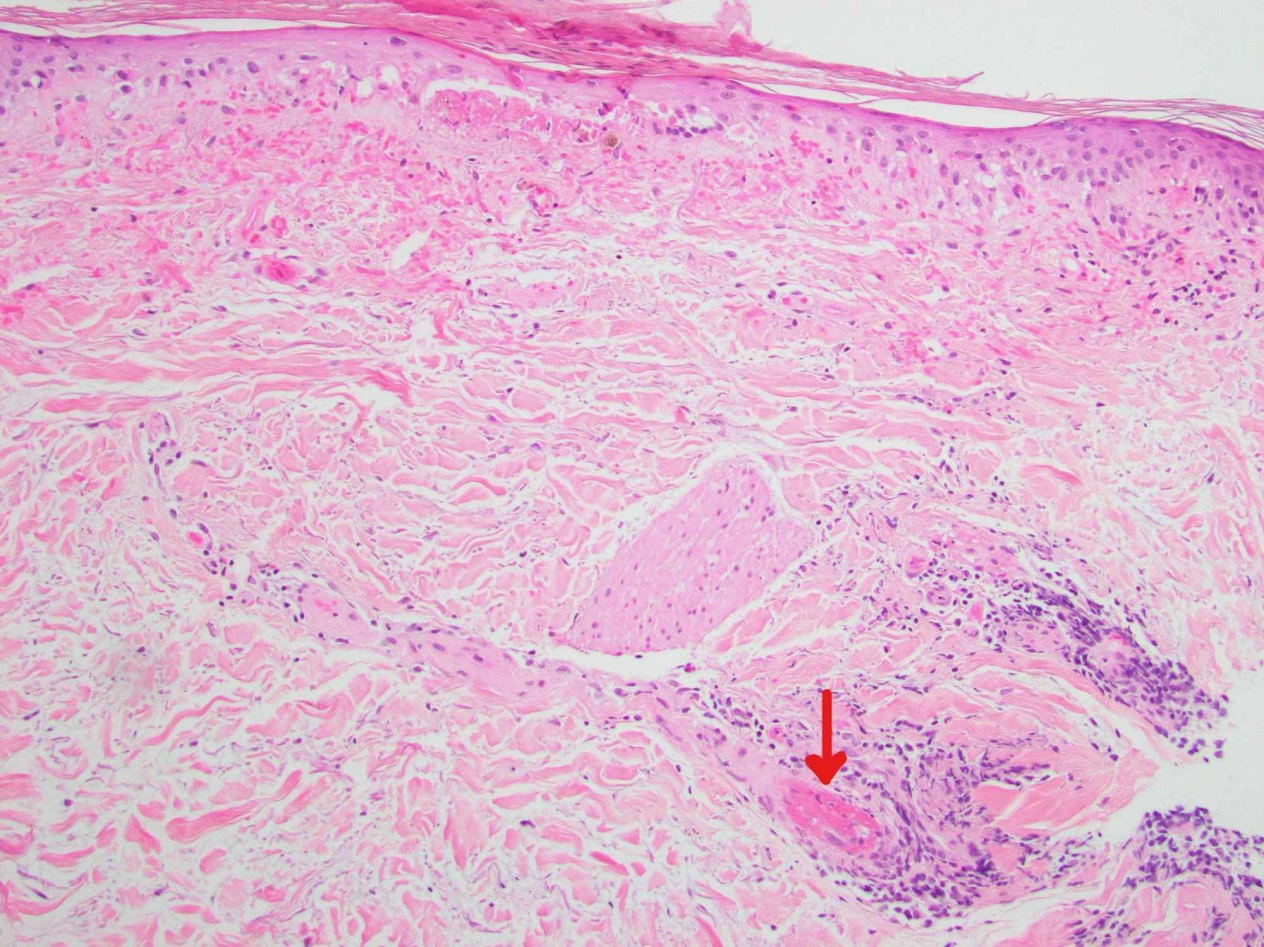
Punch biopsy of the resolving lesion (H&E ×100). The red arrow is pointing to a vessel containing luminal organizing thrombus.

**Figure 4:**
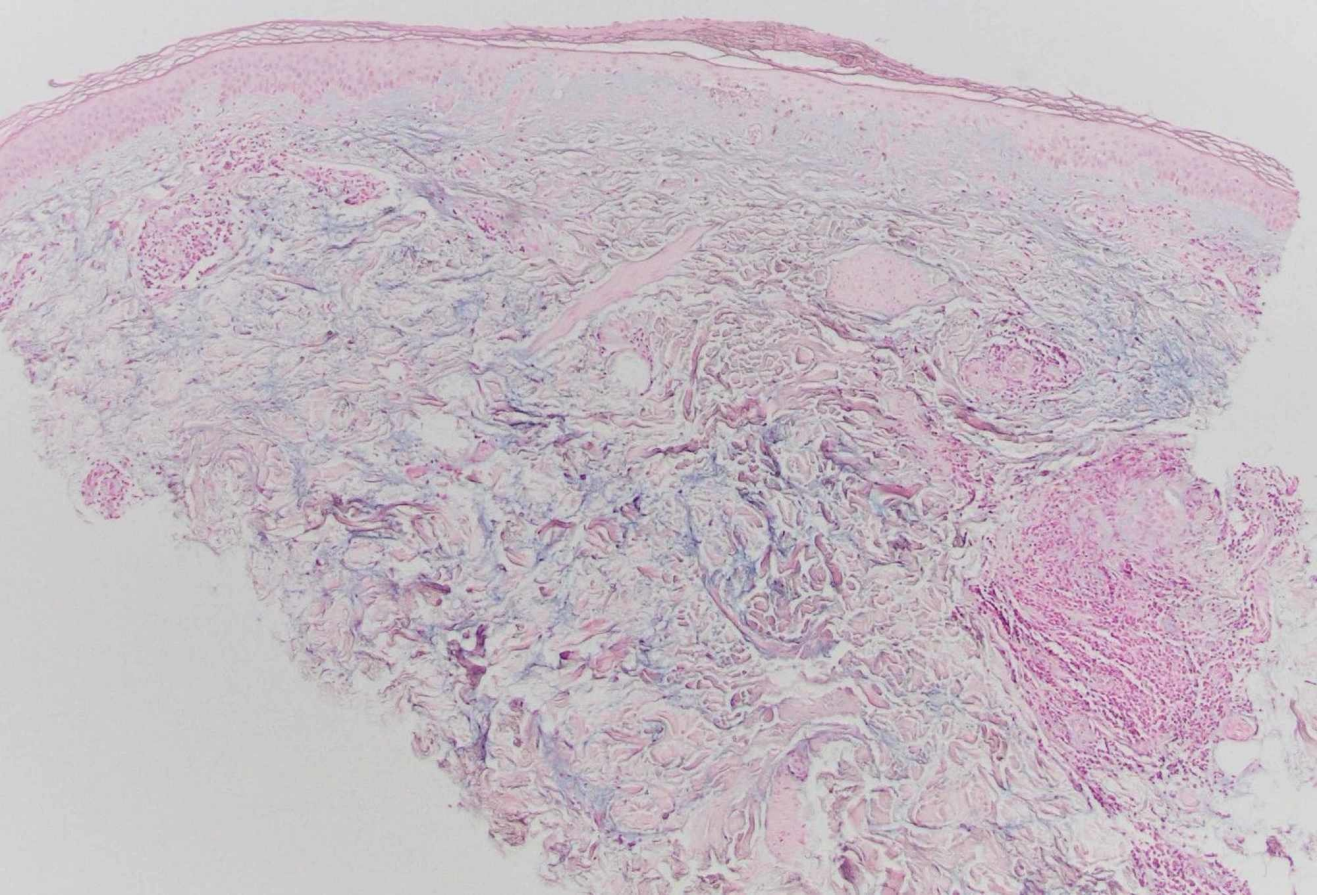
Resolving lesion (Alcian blue ×40).

Newer papules were bright pink to red, while resolving lesions were violaceous. Dermoscopy revealed visible capillaries and small vascular dots within some lesions. There were also lesions with a central white sclerotic area. The patient’s medical history included uncontrolled diabetes mellitus with diabetic ketoacidosis, infiltrating ductal carcinoma of the right breast, prior sepsis, atrial fibrillation, osteoporosis, cervical spine degeneration due to spinal stenosis, myofascial pain dysfunction syndrome, chronic abdominal pain with cramping, and chronic headaches. A biopsy was performed and submitted for pathologic examination with a clinical differential diagnosis that included angioma, telangiectasia, and capillaritis. The histologic findings showed epidermal atrophy with basement membrane thickening and vacuolar change at the dermal-epidermal junction and papillary dermal hemorrhage (Figure 2). There was markedly increased dermal mucin shown by an Alcian blue stain, as well as rare vessels containing luminal organizing thrombi (Figures 3, 4).

**Figure 5:**
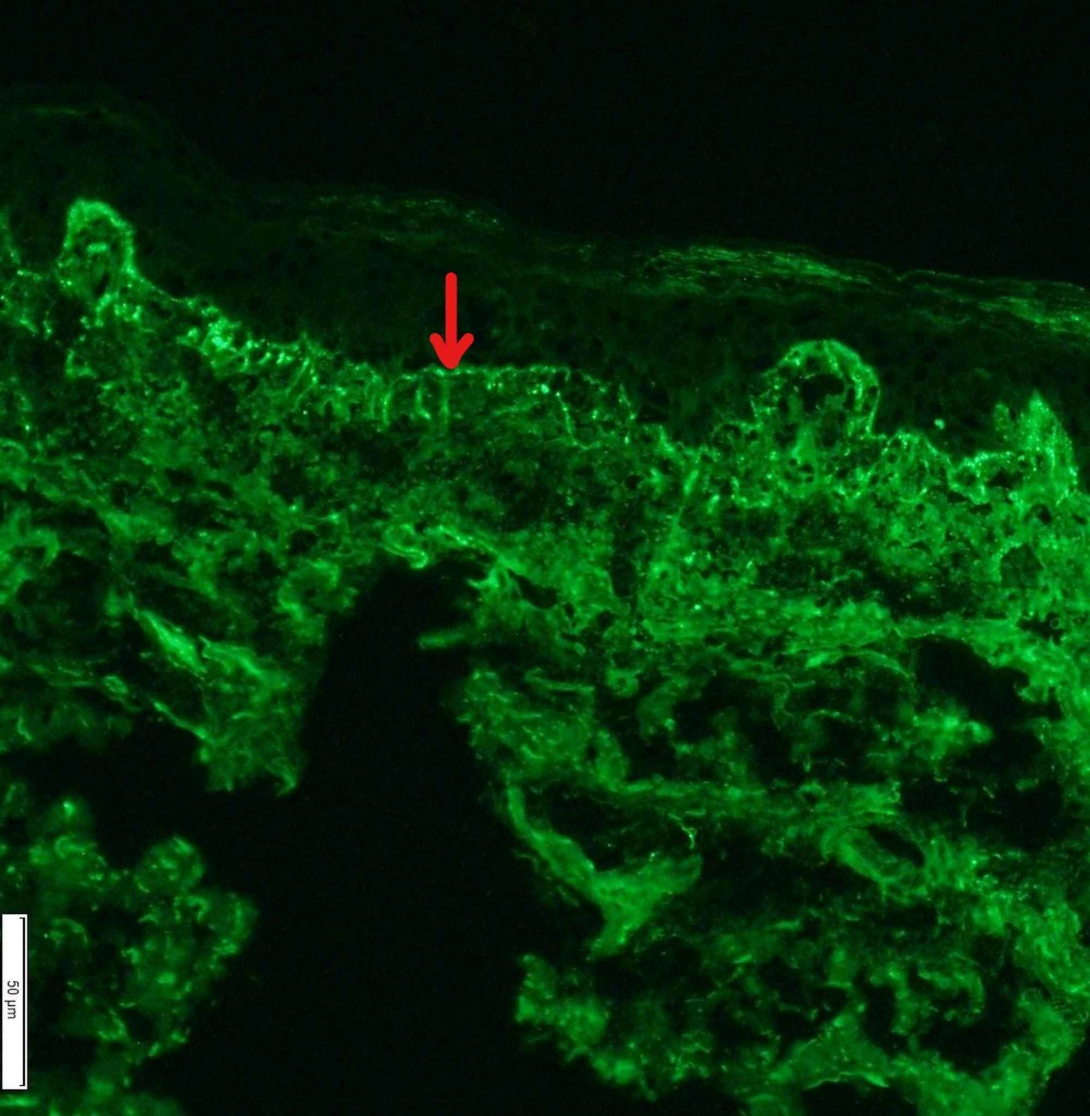
DIF (×200, IgM). The red arrow shows the strong granular staining with IgM along the dermal-epidermal junction. DIF, direct immunofluorescence

Direct immunofluorescence (DIF) revealed strong granular staining with immunoglobulin M (IgM) along the dermal-epidermal junction (Figure 5), but was negative for IgA, IgG, C3, and fibrinogen.

Overall, the histologic findings were consistent with either lupus erythematosus or Degos disease in its inflammatory stage as early Degos lesions can mimic lupus erythematosus [1]. An extensive laboratory evaluation was performed and revealed no concurrent clotting disorder (normal platelets, proteins C and S, antithrombin III, lupus anticoagulant), autoimmune condition (normal antinuclear antibody, rheumatoid factor, and anti-Smith, anti-RNP, anti-RO, anti-LA, anti-SNP, anti-dsDNA antibodies). Upper gastrointestinal endoscopy revealed mild esophagitis and gastritis. The complete eye exam showed no evidence of diabetic retinopathy or macular edema in either eye. She was also seen by a neurologist and underwent electromyography, which revealed no electrodiagnostic evidence of peripheral neuropathy. Overall, the clinical presentation was most consistent with Degos disease. In the early stages of the disease, it is often difficult to differentiate between the benign and malignant forms of Degos disease as both can present with involvement limited to the skin. To promote anticoagulation and tissue microcirculation, the patient was prescribed low-dose aspirin and pentoxifylline. She is frequently seen by her primary care physician and remains in relatively good health.

## Discussion

Although the etiology of Degos disease is obscure, it has been regarded as an endovasculitis or primary endothelial defect with secondary thrombosis leading to infarcted lesions in the skin and variably in the gastrointestinal and central nervous system [13]. The classic histopathology of a wedge-shaped area of ischemia extending from the epidermis to the reticular dermis is believed to be the result of the vaso-occlusive process [14]. Other classic findings include mild dermal-epidermal interface inflammation, scattered necrotic keratinocytes, dermal edema with copious mucin deposition, a sparse perivascular lymphocytic infiltrate, and proliferating endothelial cells with thickened vessel walls and occasional thrombosis [14,15]. Many of the histologic findings in early Degos disease mimic those found in cutaneous lupus. There is significant controversy regarding the relationship between systemic lupus erythematosus (SLE) and Degos disease. Ball et al. proposed that Degos disease is a manifestation of lupus erythematosus and not a unique disease [12]. It is also documented that patients diagnosed with SLE can develop Degos-like lesions [16,17]. However, patients without SLE have also developed Degos disease and no circulating immune complexes have yet been identified in Degos patients [13]. Because of the broad overlap in clinical features and histopathological findings between lupus and Degos disease, it is important to utilize all available diagnostic clues, including clinical and laboratory data, while evaluating these patients. DIF is a valuable addition in the diagnostic process and a useful supplement for the accurate diagnosis of cutaneous lupus erythematosus, commonly showing granular IgM (along with other immunoglobulins, complement, and fibrinogen) deposition at the dermal-epidermal junction.

In this case report, we describe the DIF finding in a patient with characteristic cutaneous lesions of Degos disease and the absence of systemic and laboratory signs of SLE. Previous reports of DIF studies performed on patients with Degos disease have shown inconsistent results, including fibrin, occasional immunoglobulins, and complement deposition near small dermal vessels or the basement membrane [14,18,19]. Granular deposition of IgM along the dermal-epidermal has not previously been reported in Degos lesions, although this DIF pattern has been described in Degos-like lesions in patients with SLE [20].

## Conclusions

In conclusion, we present a novel DIF finding that serves as additional evidence of a close relationship between Degos disease and SLE. Whether these conditions are related in terms of pathophysiology remains to be elucidated. We hypothesize that IgM deposition occurs concomitant with the basal vacuolar change that occurs in the early inflammatory phase. In patients with SLE, the presence of circulating antibodies explains the characteristic DIF patterns. In patients with Degos disease, including the one presented here, no such circulating antibodies are detected, and thus the source of antibody deposition in the skin remains unknown. While deposits of IgM in this location and pattern have been described in association with sun damage, the biopsy was not from sun-damaged skin and the intensity of the staining was far in excess of that previously described.
